# Responses in weanling pigs fed low protein diets supplemented with dietary nucleotides

**DOI:** 10.1093/tas/txae142

**Published:** 2024-10-03

**Authors:** Abiola S Lawal, Tobi Z Ogunribido, Yuechi Fu, Olayiwola Adeola, Kolapo M Ajuwon

**Affiliations:** Department of Animal Sciences, Purdue University, West Lafayette, IN 47907, USA; Department of Animal Sciences, Purdue University, West Lafayette, IN 47907, USA; Department of Animal Sciences, Purdue University, West Lafayette, IN 47907, USA; Department of Animal Sciences, Purdue University, West Lafayette, IN 47907, USA; Department of Animal Sciences, Purdue University, West Lafayette, IN 47907, USA

**Keywords:** blood urea nitrogen, growth performance, nucleotides, postweaning diarrhea

## Abstract

Evidence suggests that nucleotide supplementation in diets improves intestinal development, immune function, and cell growth. Stressful events such as weaning in pigs may increase nucleotide demand, making exogenous supplementation potentially beneficial. This study evaluated the effects of low-protein (**LP**) diets supplemented with dietary nucleotides on growth performance, postweaning diarrhea (**PWD**), nutrient digestibility, and blood metabolites. A total of 210 piglets were weaned at 21 d of age, allowing a 3-d adaptation to a common nursery diet. At 24 d, pigs were reweighed (6.02 ± 0.05 kg) and allocated to 5 dietary treatments in a randomized complete block design to give 7 replicates per treatment (*n* = 6 piglets per replicate). The 5 dietary treatments included (i) a high protein positive control diet (**PC**) with 24% crude protein (**CP**); (ii) a low protein negative control (**NC**) with 16% CP; (iii) an NC diet with nucleotide supplementation at 1 g/kg (**NC01**), 3 g/kg (**NC03**), or 9 g/kg (**NC09**). Diets were provided ad libitum for 35 d, and weekly feed intake (**FI**) and body weight (**BW**) were recorded. Blood samples were collected on day 32 and fecal samples were collected on days 33, 34, and 35 to determine serum metabolites and nutrient digestibility, respectively. Relative to PC, the NC diet had lower overall average daily gain (**ADG**) (343.5 vs. 305.5 g/d), incidence of PWD (2.5 vs. 1.2 diarrhea score), and blood urea nitrogen (**BUN**) (11.3 *vs.* 3.4 mg/dL); (*P* < 0.05, < 0.05, and < 0.0001, respectively). The nucleotide-supplemented diets, NC01, NC03, and NC09, had comparable (*P > *0.05) overall ADG to the PC and decreased (*P* < 0.0001) BUN. Additionally, NC09 had decreased (*P* < 0.05) incidence of PWD compared to PC. The apparent total tract digestibility (**ATTD**) of nitrogen increased linearly (*P* < 0.05) with nucleotide supplementation, although reducing CP decreased (*P* < 0.05) serum glutathione and insulin-like growth factor-1 (**IGF-1**) concentrations. However, IGF-1 concentration was linearly increased (*P *< 0.05) with nucleotide supplementation. Results suggest that feeding LP diets supplemented with dietary nucleotides after weaning can increase ATTD of nitrogen and protein utilization, reduce the incidence of PWD, and increase serum IGF-1 concentration while partially ameliorating the negative effects of LP diets on growth performance.

## Teaser

Dietary nucleotide supplementation of low-protein diets reduces PWD and improves growth performance and protein utilization efficiency in postweaning pigs.

## INTRODUCTION

Nucleotides and their derivatives are fundamental in biological functions, serving as major components for nucleic acids, coenzymes, and energy sources via respiratory pathways (Domeneghini et al., 2006). Cellular proliferation, tissue repair, and recovery require high nucleotide synthesis (Sanchez-Pozo and Gil, 2002). However, nucleotide synthesis consumes high energy and amino acids, such as glutamine, glycine, and aspartate. Hence, supplementation of exogenous nucleotides may result in energy and amino acids sparing, allowing these nutrients to be utilized for tissue growth (Kreuz et al., 2020).

Under normal conditions, de novo synthesis of nucleotides and recovery via salvage pathway meet the nucleotide requirement of mammals (Hess and Greenberg, 2012). However, conditions such as weaning in pigs heighten nucleotide requirements due to stress, rapid growth rate, and compromised immune system (Valini et al., 2021). These result in the impairment of energy and nucleic acid synthesis, making de novo synthesis of nucleotides metabolically expensive (Domeneghini et al., 2006). Therefore, the provision of dietary nucleotides becomes crucial in these circumstances, as nucleotides become conditionally essential (Domeneghini et al., 2006; Valini et al., 2021). Notably, the gut has limited capability for de novo nucleotide synthesis, relying heavily on the salvage pathway for nucleotide synthesis (Grimble, 1994). Indeed, supplementing diets with ingredients, such as yeast-derived nucleotides, has been reported to confer additional benefits on pigs, including improved gut structure, intestinal barrier function, and immune response, increased cell proliferation, and decreased incidence of PWD in pigs (Domeneghini et al., 2006; Martinez-Puig et al., 2007; Jang and Kim, 2019; Valini et al., 2021).

During the initial 2 weeks after weaning, pigs are highly susceptible to postweaning diarrhea **(PWD)**, which is characterized by watery feces, dehydration, weight loss, and death in extreme cases (Heo et al., 2013; Rhouma et al., 2017). Postweaning diarrhea typically results from the proliferation of one or more strains of enterotoxigenic *Escherichia coli***(ETEC)**, notably the F4 (K88+) and F18 + *Escherichia coli*, in the gastrointestinal tract (**GIT**). This has an adverse effect on the gut health of pigs as it destroys the integrity of intestinal barriers and induces inflammation in newly weaned pigs (Kim et al., 2022). Disturbance in gut function at weaning results in major economic losses in pig production due to decreased growth rate, increased expenditure on antibiotics, and losses from mortality (Kim et al., 2022; Jerez-Bogota et al., 2023).

According to the NRC (2012) guidelines, postweaning pigs require 3.63 g/kg of N, corresponding to 22.7% of dietary crude protein (CP), to maintain optimal growth performance. However, this level exceeds the digestive capability of most weanling pigs fed many of the common feed ingredients, leading to the accumulation of undigested dietary protein in the hindgut, increased fermentation, and production of potentially toxic substances and environmental pollution due to excess N excretion (Heo et al., 2010; Yu et al., 2019; Kim et al., 2023). End products of protein fermentation include indoles, phenols, ammonia, and amines that contribute to the development of PWD by impairing gut function and increasing the proliferation of pathogens (Rist et al., 2013; Heo et al., 2015; Gilbert et al., 2018). The quantity of undigested proteins reaching the hindgut depends on both the intake level and the source of the proteins (Gilbert et al., 2018). Consequently, using low-protein (**LP**) diets in nursery pig production is considered a highly effective approach to reducing N excretion and PWD (Heo et al., 2010; Wang et al., 2018; Yu et al., 2019).

However, various studies have reported that a substantial reduction in dietary CP levels may impair the growth performance of pigs primarily due to deficiencies in the abundance of essential or nonessential amino acids and resulting in morphological alterations such as a decrease in villous height observed in the duodenum and jejunum (Nyachoti et al., 2006; Deng et al., 2009; Spring et al., 2020a; Batson et al., 2021). To ameliorate the negative effects of crude protein **(CP)** reduction in diets, LP diets are typically supplemented with crystalline amino acids. For instance, the five essential amino acids (lysine, methionine, threonine, tryptophan, and valine) are generally available in crystalline form for use in diet formulation, and additional reductions in dietary CP can be achieved by meeting the amino acid requirements of pigs (Rocha et al., 2022). Although nucleotide supplementation has potential to spare amino acids use for de novo nucleotide synthesis, the effect of this on animal performance in the context of an LP diet has not been previously investigated.

Therefore, we hypothesized that nucleotide supplementation of LP diets would ameliorate the negative effects of CP reduction on growth performance while reducing the incidence of PWD and mitigating N excretion and release to the environment. Thus, the objective of this study was to investigate the effects of nucleotide supplementation to LP diets on growth performance, incidence of PWD, blood metabolites, nutrient digestibility, and protein utilization in weanling pigs.

## MATERIALS AND METHODS

All animal procedures used in this study were reviewed and approved by the Purdue University Animal Care and Use Committee (Approval No. 1111000145).

### Animals, diets, and experimental Design

A total of 210 piglets (Landrace × (either Yorkshire or Duroc) were weaned at 21 d of age and fed ad libitum, a common corn-soybean nursery diet, during a 3-d adaptation. At 24 d, pigs were reweighed and allocated to 5 dietary treatments in a randomized complete block design. The initial body weight was 6.02 ± 0.05 kg, and the experimental period lasted for 35 d. Each dietary treatment consisted of 7 replicate pens containing 6 piglets per replicate (3 gilts and 3 barrows).

Nucleotides (Nucleosaf 600; Phileo by Lesaffre, Marcq-en-Baroeul, France) were added to the nucleotide-supplemented diets. The 5 dietary treatments were as follows: (i) a high protein positive control diet **(PC)** with 24% crude protein (CP); (ii) a low protein negative control **(NC)** with 16% CP; (iii) NC with 1 g/kg dietary nucleotide inclusion **(NC01)**; (iv) NC with 3 g/kg dietary nucleotide inclusion **(NC03)**; and (v), NC with 9 g/kg dietary nucleotide inclusion **(NC09)**. The chemical composition of the nucleotide supplement is outlined in Supplementary Table S1. All diets were corn-soybean meal-based, supplemented with lysine, methionine, tryptophan, and threonine, with all the low-protein (**LP**) diets additionally fortified with crystalline isoleucine and valine to meet the amino acid requirements outlined in NRC (2012). On day 28, titanium dioxide was added at 5 g/kg to all diets as an indigestible marker to assess the apparent total tract digestibility (**ATTD**) of nutrients. Pigs had ad libitum access to feed and water for the 35 d of the experiment. The dietary compositions are presented in Table 1, which includes the contribution of essential amino acids from the nucleotide-containing product used.

**Table 1.** Ingredient composition of experimental diets, as-fed basis

Ingredients, g/kg	PC	NC	NC01	NC03	NC09
Ground corn	440.20	624.49	604.49	562.49	440.20
DDGS	50.00	50.00	50.00	50.00	50.00
Wheat bran	20.00	20.00	20.00	20.00	20.00
Soybean meal, 48% CP	350.00	144.00	144.00	144.00	144.00
Soybean oil	35.00	42.00	42.00	44.00	46.29
Soy protein concentrate	41.20	40.00	40.00	40.00	40.00
Ground limestone	15.00	15.00	15.00	15.00	15.00
Monocalcium phosphate	12.00	14.50	14.50	14.50	14.50
Salt	4.00	4.00	4.00	4.00	4.00
l-Lysine HCl	2.00	8.74	8.74	8.74	8.74
dl-Methionine	0.80	1.55	1.55	1.55	1.55
l-Threonine	0.40	3.00	3.00	3.00	3.00
l-Tryptophan	0.20	0.60	0.60	0.60	0.60
l-Isoleucine	0.25	1.27	1.27	1.27	1.27
l-Valine	0.25	2.15	2.15	2.15	2.15
Vitamin premix^1^	2.50	2.50	2.50	2.50	2.50
Mineral premix ^2^	0.70	0.70	0.70	0.70	0.70
Selenium premix^3^	0.50	0.50	0.50	0.50	0.50
Nucleotide premix^4^	0.00	0.00	20.00	60.00	180.00
Titanium dioxide^5^	25.00	25.00	25.00	25.00	25.00
Total	1000.00	1000.00	1000.00	1000.00	1000.00
Calculated composition, g/kg					
ME, kcal/kg	3354.18	3356.72	3353.32	3356.89	3348.38
CP	236.69	163.82	163.73	163.40	162.72
Ca	8.80	8.57	8.57	8.57	8.57
P	6.66	6.20	6.20	6.19	6.17
Phytate-P	2.51	2.10	2.10	2.09	2.08
STTD P	4.08	4.01	4.01	4.00	4.00
Analyzed composition, g/kg					
GE, kcal/kg	4.09	4.07	4.03	4.08	4.08
CP	221.6	179.5	171.8	179.7	173.6
Ca	11.17	11.97	11.30	11.22	10.21
P	6.25	6.67	6.72	6.20	6.23
Calculated standard ileal digestible amino acid composition, g/kg					
Indispensable AA					
Arg	15.19	9.05	9.07	9.10	9.20
His	5.87	3.84	3.85	3.85	3.88
Ile	9.50	6.98	6.99	7.02	7.11
Leu	18.31	13.23	13.24	13.25	13.31
Lys	13.44	13.62	13.68	13.77	14.08
Met	4.13	3.92	3.92	3.93	3.95
Met + Cys	7.42	6.26	6.27	6.27	6.30
Phe	10.71	6.93	6.99	7.09	7.41
Thr	8.19	7.88	7.90	7.92	8.00
Trp	2.90	2.14	2.15	2.16	2.20
Val	10.17	8.59	8.61	8.65	8.76
Dispensable AA					
Ala	12.56	9.38	9.37	9.34	9.25
Asp	26.14	15.90	15.89	15.86	15.78
Cys	4.10	3.00	2.99	2.98	2.95
Glu	38.66	21.20	21.19	21.18	21.16
Gly	10.40	6.84	6.84	6.82	6.77
Pro	14.65	10.71	10.69	10.65	10.55
Ser	12.33	8.13	8.13	8.10	8.05
Tyr	8.40	5.57	5.57	5.55	5.51

^1^Provided the following quantities per kilogram of complete diet: vitamin A, 6,600 IU; vitamin D_3_, 1,660 IU; vitamin E, 44 IU; menadione, 2.2 mg; riboflavin, 8.8 mg; D-pantothenic acid, 22 mg; niacin, 33 mg; and vitamin B_12_, 0.04 mg.

^2^Provided the following quantities per kilogram of complete diet: I, 0.26 mg; Mn, 12.0 mg; Cu, 6.33 mg; Fe, 136 mg; and Zn, 104 mg.

^3^Provided 0.3 mg Se/kg of complete diet.

^4^Nucleotides product was added to diets as a replacement for corn in a premix to supply added at 0, 1, 3, or 9 g/kg of complete diet.

^5^Provided 5 g TiO_2_/kg of complete diet.

### Sample collection and processing

Weekly measurements of body weight (**BW**) and feed intake (**FI**) were taken over the 35-d period to determine the average daily gain (**ADG**), average daily feed intake (**ADFI**), and gain-to-feed ratio (**G:F**). Fresh fecal samples were collected from all pigs in each replicate pen on days 33, 34, and 35 by rectal palpation to determine the apparent total tract digestibility (**ATTD**) of nutrients. Feces were collected by hand immediately as the pigs began to defecate. All fecal samples were immediately stored at −20°C until subsequent analysis.

On day 32, blood samples (10 mL) were collected by jugular venipuncture from the pig closest to the average BW of the pen using nonheparinized vacutainer tubes (Monoject blood collection tube, Covidien, Minneapolis, MN, USA). Serum was obtained by centrifuging blood samples 3,000 × *g* for 15 min at 4°C. The serum was then aspirated and stored at −80°C until further analyses. To determine the frequency and severity of diarrhea, a scoring system ranging from 1 to 4 (1 = normal feces, 2 = soft feces, 3 = mild diarrhea, and 4 = severe diarrhea) was established. Two independent evaluators visually assessed each pen daily and recorded scores, spanning from days 0 to 14 (Alagbe et al., 2022).

### Determination of apparent total tract digestibility of energy, dry matter, phosphorus, calcium, and nitrogen

Fecal subsamples were pooled within each replicate pen and oven-dried at 55°C until a constant weight was achieved. Experimental diets and dried fecal samples were ground using a 0.5 mm sieve and oven-dried at 105°C for 24 h to determine the dry matter (**DM**) content (Precision Scientific Co., Chicago, IL; method 934.01; AOAC, 2006). Gross energy (**GE**) in samples was determined using a bomb calorimeter (Parr 1261 bomb calorimeter, Parr Instrument Co., Moline, IL), and nitrogen (**N**) content was analyzed using the combustion method (TruMac N; LECO Corp., St. Joseph, MI; method 990.03; AOAC, 2000). To determine the concentration of phosphorus (**P**) in samples, diets, and fecal samples were ashed in a muffle furnace (Fisher Scientific Isotemp, St. Louis, MO, USA) at 600°C for 16 h. The resulting ash was digested in a solution containing 20 mL of 4 M HCl and 5 drops of concentrated nitric acid, heated for approximately 7 minutes on a hot plate, and then transferred into 250 mL volumetric flasks. Phosphorus levels were determined through spectrophotometric analysis (AOAC International, 2006) on a TECAN Spark multimode microplate reader (Männedorf, Switzerland), measuring absorbance at 620 nm as described by Zhai and Adeola (2013). Titanium concentration was measured following the procedure outlined by Myers et al. (2004), and calcium (**Ca**) concentration was determined using an inductively coupled plasma-optical emission spectrometer (Varian, Model 720-ES) according to method 968.08 (AOAC International, 2000).

### Serum analysis

The concentrations of serum insulin-like growth factor 1 (**IGF-1**), insulin, glucose, and blood urea nitrogen (**BUN**) were measured using commercial kits and procedures as recommended by the respective manufacturers: IGF-1 (RayBio Porcine IGF-1 ELISA Kit, RayBiotech, Norcross, GA, USA); insulin (Mercodia Porcine Insulin ELISA, Mercodia, Winston-Salem, NC, USA); glucose (Autokit Glucose, Fujifilm, Lexington, MA, USA) and BUN (Urea Nitrogen Colorimetric Detection Kit, Thermo Fisher Scientific, Frederick, MD, USA). The serum concentration of glutathione (**GSH**) was measured according to protocols described by Rahman et al. (2006).

### Calculations and statistical analysis

The ATTD % of nutrients in both the experimental diets and fecal samples were calculated using the equations described by Adeola (2001):


$$\begin{array}{l} \left[ATTD,\;\%=100-\left(\frac{T_{ii}}{T_{io}}\right)\times \left(\frac{Y_{o}}{Y_{i}}\right)\times 100\right] \end{array}$$

where *T*_*ii*_ and *T*_*io*_ are the titanium concentrations of the diets and fecal samples, respectively (g/kg DM); and *Y*_*o*_ and *Y*_*i*_ are the concentrations of GE, P, Ca, and N in the fecal samples and diets, respectively (g/kg DM).

Data points beyond ± 1.5 × interquartile range from the dataset’s median were identified and excluded as outliers. Growth performance, blood characteristics, and nutrient digestibility were analyzed by PROC GLM of SAS (v.9.4; SAS Inst. Inc., Cary, NC). Comparisons between diets were performed using the orthogonal contrast analysis. Specifically, the PC was compared to each of the NC diets, whereas the NC was compared to each of the nucleotide-supplemented NC diets. The contrast was used as A) PC vs. NC, B) PC vs. NC01, C) PC vs. NC03, D) PC vs. NC09, E) NC vs. NC01, F) NC vs. NC03, and G) NC vs. NC09. Each pen was considered as an experimental unit, and the experimental diets were considered as fixed effects. Replicate blocks were regarded as random effects in a randomized complete block design. The contrast coefficients necessary to assess the linear and quadratic effects of increasing nucleotide levels were derived using the IML procedure of SAS. The frequency of diarrhea data was analyzed using the LOGISTIC procedure of SAS. Prior to using this procedure, data were expressed as the proportion of diarrhea score ≥2 divided by the overall number of pens (Alagbe et al., 2022). Variability within the data was represented by the pooled standard error of the mean, and statistical significance was declared at *P* ≤ 0.05, whereas a tendency was declared at 0.05 < *P* ≤ 0.10.

## RESULTS

### Nucleotide supplementation partially alleviated the reduction in growth performance

Compared to PC, NC had decreased overall ADG (*P* < 0.05, Table 2). However, the overall ADFI and G:F did not differ between the two diets. Compared to PC, the nucleotide-supplemented groups showed a tendency for decreased overall ADFI (*P* = 0.06). However, the overall ADG, final BW, and G:F were not different between the nucleotide-supplemented groups and PC. Also, the nucleotide-supplemented groups did not differ in overall ADG and final BW from the NC. Pigs fed dietary nucleotides had a tendency (*P* = 0.08) for a linear increase in ADG from days 7 to 14 and a linear increase in ADG from days 21 to 28 (*P* = 0.05). From days 21 to 28, there was a quadratic effect (*P* < 0.05) of nucleotide supplementation on G:F as the NC03 group had the lowest G:F followed by NC01, NC, and NC09. NC09 tended to have increased G:F relative to NC from days 0 to 7 (*P *= 0.08) and overall G:F (*P *= 0.09) from days 0 to 35.

**Table 2.** Growth performance of weanling pigs fed low protein diets supplemented with dietary nucleotides from days 0 to 35^1^

Diet	PC	NC	NC01	NC03	NC09	Pooled SEM^2^	Contrasts^3^							*P*-value	
NUCL, %	0	0	0.1	0.3	0.9										
Items							A	B	C	D	E	F	G	Linear	Quadratic
BW, kg															
Day 0	6.07	6.06	6.06	5.97	5.96	0.05	0.91	0.86	0.15	0.11	0.95	0.18	0.13	0.08	0.32
Day 7	6.29	6.19	6.27	6.05	6.19	0.08	0.38	0.91	0.04	0.40	0.45	0.21	0.98	0.82	0.21
Day 14	7.27	7.10	7.14	6.97	7.40	0.16	0.45	0.58	0.19	0.55	0.83	0.57	0.18	0.12	0.24
Day 21	10.06	9.71	9.67	9.62	10.11	0.26	0.34	0.30	0.24	0.90	0.93	0.81	0.28	0.22	0.52
Day 28	13.14	12.98	13.11	13.00	13.68	0.32	0.73	0.96	0.76	0.24	0.77	0.97	0.13	0.12	0.61
Day 35	17.91	16.81	17.13	16.98	17.55	0.40	0.06	0.18	0.11	0.54	0.57	0.76	0.20	0.24	0.90
ADG, g/d															
Day 0-7	34.40	19.53	30.95	20.28	36.54	8.77	0.25	0.79	0.29	0.87	0.35	0.95	0.17	0.23	0.69
Days 7-14	156.02	122.84	129.26	145.57	162.87	18.24	0.22	0.32	0.71	0.80	0.80	0.39	0.12	0.08	0.61
Days 14-21	411.68	378.37	358.73	364.11	377.19	24.38	0.35	0.14	0.21	0.33	0.56	0.69	0.97	0.82	0.72
Days 21-28	434.08	454.49	456.84	457.43	503.78	18.76	0.45	0.41	0.42	0.02	0.93	0.91	0.07	0.05	0.59
Days 28-35	720.41	546.48	591.53	602.92	561.11	33.90	<0.01	0.01	0.03	<0.01	0.34	0.26	0.75	0.86	0.17
Days 0-35	343.47	305.47	317.35	320.31	324.15	10.97	0.02	0.11	0.17	0.23	0.43	0.35	0.22	0.31	0.62
ADFI, g/d															
Days 0-7	163.53	163.04	161.56	148.57	160.51	8.29	0.97	0.87	0.24	0.28	0.90	0.24	0.28	0.30	0.40
Days 7-14	278.45	260.11	238.10	251.86	305.00	20.01	0.72	0.22	0.39	0.30	0.35	0.58	0.15	0.03	0.25
Days 14-21	575.54	570.87	481.51	552.70	532.48	35.56	0.90	0.07	0.77	0.34	0.04	0.67	0.26	0.75	0.94
Days 21-28	935.75	944.06	975.21	1070.44	982.12	53.60	0.92	0.61	0.07	0.45	0.67	0.07	0.49	0.64	0.10
Days 28-35	1188.33	927.70	983.78	977.90	987.41	52.81	0.00	0.02	0.03	0.01	0.44	0.34	0.45	0.61	0.47
Days 0-35	603.08	576.22	565.30	567.74	568.47	12.03	0.13	0.06	0.06	0.06	0.51	0.63	0.64	0.85	0.69
G:F, g/kg															
Days 0-7	211.73	119.18	191.13	136.16	227.49	52.18	0.15	0.79	0.31	0.84	0.21	0.68	0.08	0.14	0.89
Days 7-14	559.68	473.90	545.30	577.82	531.72	51.04	0.25	0.85	0.82	0.71	0.31	0.17	0.41	0.69	0.21
Days 14-21	714.50	664.71	744.14	658.72	708.52	35.22	0.33	0.56	0.30	0.91	0.11	0.91	0.37	0.78	0.74
Days 21-28	463.55	481.13	468.54	427.40	513.55	24.36	0.47	0.89	0.33	0.17	0.54	0.09	0.47	0.26	0.04
Days 28-35	605.59	589.51	601.74	617.72	571.77	28.52	0.70	0.93	0.79	0.42	0.76	0.50	0.65	0.49	0.37
Days 0-35	569.02	530.93	561.28	566.93	570.56	16.41	0.12	0.74	0.93	0.95	0.19	0.14	0.09	0.21	0.34

^1^PC = positive control diet with adequate CP; NC = negative control diet with inadequate CP; NC01 = negative control diet containing dietary nucleotides at 1.0 g/kg of diet; NC03 = negative control diet containing dietary nucleotides at 3.0 g/kg of diet; NC09 = negative control diet containing dietary nucleotides at 9.0 g/kg of diet; NUCL = nucleotides; BW = body weight; ADG = average daily gain; ADFI = average daily feed intake; G:F = gain-to-feed ratio.

^2^Pooled SEM, pooled standard error of mean.

^3^Contrasts: A) PC vs. NC; B) PC vs. NC01; C) PC vs. NC03; D) PC vs. NC09; E) NC vs. NC01; F) NC vs. NC03; and G) NC vs. NC09.

### Low-protein diets reduced the incidence of postweaning diarrhea

A reduction in the overall diarrhea frequency (days 0 to 14) was observed in piglets fed NC diet relative to PC (*P* < 0.05, Table 3). Also, the NC09 group had decreased overall diarrhea frequency relative to PC (*P* < 0.05), while there was no difference relative to NC. There were no linear or quadratic trends of nucleotide supplementation on the overall diarrhea frequency.

**Table 3.** The overall diarrhea frequency of weanling pigs fed low protein diets supplemented with dietary nucleotides from days 0 to 14^1^

Diet	PC	NC	NC01	NC03	NC09	SD^2^	Contrasts^3^							*P*	
NUCL, %	0	0	0.1	0.3	0.9										
Diarrhea frequency							A	B	C	D	E	F	G	Linear	Quadratic
Days 0-14	2.46	1.20	1.69	1.57	1.34	0.39	0.03	0.26	0.60	0.03	0.33	0.16	0.91	0.40	0.64

^1^PC = positive control diet with adequate CP; NC = negative control diet with inadequate CP; NC01 = negative control diet containing dietary nucleotides at 1.0 g/kg of diet; NC03 = negative control diet containing dietary nucleotides at 3.0 g/kg of diet; NC09 = negative control diet containing dietary nucleotides at 9.0 g/kg of diet; NUCL = nucleotides.

^2^SD, standard deviation.

^3^Contrasts: A) PC vs. NC; B) PC vs. NC01; C) PC vs. NC03; D) PC vs. NC09; E) NC vs. NC01; F) NC vs. NC03; and G) NC vs. NC09.

### Effects of nucleotide supplementation on apparent total tract digestibility of nutrients

Compared to PC, NC03 had decreased ATTD of DM (*P* < 0.05, Table 4), while NC01 showed a tendency (*P* = 0.09) for a decrease. The NC01 and NC03 groups had decreased ATTD of GE (*P* < 0.05), while NC09 was not different from PC, potentially indicating nucleotide effect at this level of supplementation. There were no differences among the LP diets for ATTD of GE, irrespective of nucleotide supplementation. For the ATTD of N, the nucleotide-supplemented groups were not different from PC, except for NC03 which showed a tendency for a higher N digestibility (*P* = 0.07). There was a linear increase in ATTD of N in the nucleotide-supplemented groups (*P* < 0.05). No treatment effects were observed for ATTD of Ca and P.

**Table 4.** Apparent total tract digestibility (ATTD) of nutrients in weanling pigs fed low protein diets supplemented with dietary nucleotides^1^

Diet	PC	NC	NC01	NC03	NC09	Pooled SEM^2^	Contrasts^3^							*P*	
NUCL, %	0	0	0.1	0.3	0.9		A	B	C	D	E	F	G	Linear	Quadratic
ATTD, %															
DM	83.12	82.25	81.91	81.33	82.14	0.49	0.22	0.09	0.01	0.16	0.63	0.19	0.87	0.93	0.19
GE	81.32	80.08	79.03	79.09	80.12	0.49	0.10	<0.01	<0.01	0.10	0.15	0.17	0.96	0.48	0.15
Ca	66.57	64.66	67.46	65.49	66.60	1.20	0.27	0.61	0.53	0.99	0.11	0.63	0.26	0.59	0.85
P	46.20	43.92	44.84	43.28	44.20	1.96	0.42	0.62	0.31	0.46	0.75	0.83	0.92	0.43	0.85
N	75.26	74.33	76.98	77.51	76.54	0.91	0.48	0.16	0.07	0.29	0.05	0.02	0.09	0.02	0.39

^1^PC = positive control diet with adequate CP; NC = negative control diet with inadequate CP; NC01 = negative control diet containing dietary nucleotides at 1.0 g/kg of diet; NC03 = negative control diet containing dietary nucleotides at 3.0 g/kg of diet; NC09 = negative control diet containing dietary nucleotides at 9.0 g/kg of diet; NUCL = nucleotides, DM = dry matter; GE = gross energy; Ca = calcium; P = phosphorus; N = nitrogen.

^2^Pooled SEM, pooled standard error of mean.

^3^Contrasts: A) PC vs. NC; B) PC vs. NC01; C) PC vs. NC03; D) PC vs. NC09; E) NC vs. NC01; F) NC vs. NC03; and G) NC vs. NC09.

### Low-protein diets reduced BUN, serum GSH, and IGF-1 but had no effects on serum glucose and insulin

Serum concentrations of BUN and GSH decreased with reduced CP level (Table 5) without an effect of nucleotide supplementation (*P* < 0.0001 and < 0.05, respectively). Serum concentration of IGF-1 decreased with reduced CP level (*P* < 0.05), except for NC09, which did not differ from PC. There was a linear increase in serum IGF-1 concentration with nucleotide supplementation (*P* < 0.05). No treatment effects were observed for serum glucose and insulin concentrations.

**Table 5.** Blood serum characteristics of weanling pigs fed low-protein diets supplemented with dietary nucleotides^1^

Diet	PC	NC	NC01	NC03	NC09	Pooled SEM^2^	Contrasts^3^							*P*-value	
NUCL, %	0	0	0.1	0.3	0.9										
Items							A	B	C	D	E	F	G	Linear	Quadratic
GLU, mg/dL	179.68	166.51	157.65	168.81	171.42	8.93	0.30	0.12	0.39	0.55	0.48	0.84	0.70	0.43	0.98
INS, pmol/L	48.99	54.34	52.72	54.90	59.18	7.97	0.66	0.76	0.62	0.40	0.96	0.96	0.66	0.59	0.91
BUN, mg/dL	11.33	3.40	3.25	3.00	2.68	0.75	<0.0001	<0.0001	<0.0001	<0.0001	0.88	0.70	0.50	0.36	0.82
GSH, μmol/L	12.17	7.77	7.76	8.16	7.65	1.02	0.01	0.01	0.01	0.01	1.00	0.79	0.93	0.91	0.72
IGF-1, ng/mL	237.65	215.69	218.64	218.87	228.10	5.66	0.01	0.04	0.02	0.25	0.72	0.67	0.12	0.04	0.86

^1^PC = positive control diet with adequate CP; NC = negative control diet with inadequate CP; NC01 = negative control diet containing dietary nucleotides at 1.0 g/kg of diet; NC03 = negative control diet containing dietary nucleotides at 3.0 g/kg of diet; NC09 = negative control diet containing dietary nucleotides at 9.0 g/kg of diet; NUCL = nucleotides; GLU = glucose; INS = insulin; BUN = blood urea nitrogen; GSH = glutathione; IGF-1 = insulin-like growth factor-1.

^2^Pooled SEM, pooled standard error of mean.

^3^Contrasts: A) PC vs. NC; B) PC vs. NC01; C) PC vs. NC03; D) PC vs. NC09; E) NC vs. NC01; F) NC vs. NC03; and G) NC vs. NC09.

## DISCUSSION

Nucleotides, the monomeric constituents of nucleic acid polymers of DNA, RNA, and ATP, have garnered considerable attention due to their beneficial effects on immune response, intestinal development, and growth performance, particularly in postweaning pigs (Weaver and Kim, 2014). Prior to weaning, piglets obtain their nucleotide intake from sow milk (Perricone et al., 2020). However, postweaning diets often lack sufficient concentrations of nucleotides (Weaver and Kim, 2014; Perricone et al., 2020). The importance of sufficient nucleotide supplementation is especially critical in the gut, which has a limited capacity for de novo nucleotide synthesis (Grimble, 1994). Additionally, weaning stress creates a heightened demand for nucleotides, making them conditionally essential during this period (Correa et al., 2021). Therefore, the current study in which nucleotides were supplemented to a low-protein diet at various levels represents a novel perspective on the potential of supplemental nucleotides to support pig growth performance.

In the current study, no treatment effects were observed between days 0 and 7. This is probably due to postweaning lag as pigs were getting acclimatized to the new feed and environment. The reduction in overall ADG and tendency for a decreased final BW in the NC diet despite supplementation with branched-chain amino acids is consistent with previous findings indicating that LP diets decreased growth performance, irrespective of supplementation with few limiting amino acids (Yue and Qiao, 2008; Spring et al., 2020b). Despite the addition of supplemental amino acids to the LP diets, there were still deficiencies in phenylalanine and histidine, and this could have contributed to the lower growth performance in the LP diets in general. However, supplementation with nucleotides tended to linearly increase ADG between days 7 and 14 and increased ADG between days 21 and 28, with the greatest increase observed in the 0.9% nucleotide-supplemented group. This is consistent with previous findings indicating that nucleotide supplementation in nursery pigs resulted in a linear increase in ADG and other performance improvements (Weaver and Kim, 2014; Perricone et al., 2020). Overall, nucleotide supplementation partly ameliorated the negative effects of LP diets on growth performance. However, our findings contradict some previous studies, such as those conducted by Lee et al. (2007); Sauer et al. (2012); and Correa et al. (2021), which reported no improvement in growth performance following nucleotide supplementation. Specifically, Lee et al. (2007) administered dietary nucleotides at a concentration of 0.1% over a 28-d period, whereas Sauer et al. (2012) and Correa et al. (2021) employed an oral administration of nucleotides. The discrepancies in findings could be due to differences in diet composition between experiments, length of study, supplementation methods, sources, and dosage of nucleotides. However, the reduction in ADFI with nucleotide supplementation was unexpected and is in contrast to the findings by Perricone et al. (2020) and Weaver and Kim (2014), who reported that nucleotide supplementation increased ADFI in postweaning pigs. Similar to the findings of Perricone et al. (2020), G:F was not improved by nucleotide supplementation in the NC01 group (0.57 g/head/d of nucleotides) and the NC03 group (1.70 g/head/d of nucleotides). Perricone et al. (2020) reported that daily oral administration of nucleotides (0.8 g/head/d of mixture of nucleotides) for 28 d did not increase G:F. The exception was the NC09 group (5.12 g/head/d of nucleotides), which showed a tendency for a higher G:F compared to NC, suggesting that a higher level of nucleotide supplementation may be necessary for it to affect G:F. Future experiments where higher levels of nucleotide supplementation are used may be necessary to answer this question.

Diarrhea typically occurs within the first 2 weeks of weaning due to multiple factors which include changes to the diet and housing, immature digestive and immune system, among others (Rhouma et al., 2017). The reduction in postweaning diarrhea (**PWD**) observed in this study, alongside a decrease in CP may indicate that reducing dietary and digesta N content may result in subsequent decrease in undigested N entering the hindgut, reducing the chances of microbial fermentation and proliferation of potentially pathogenic microorganisms and PWD (Heo et al., 2010; Lynegaard et al., 2021). This result agrees with other studies in which LP diets reduced the incidence of diarrhea in early postweaning period (Heo et al., 2010, 2013; Lynegaard et al., 2021). Additionally, in pigs supplemented with 0.9% nucleotides, the lower incidence of PWD compared to PC may be attributed to both the impact of a low-protein (LP) diet and the additive influence of dietary nucleotides. A previous study found that 1 and 2 g/kg nucleotide supplementation reduced PWD, as evidenced by a decrease in the number of pigs treated with antibiotic as a result of PWD (Martinez-Puig et al., 2007). Additionally, Weaver and Kim (2014) reported that nucleotides high in inosine 5´ monophosphate reduced the incidence of PWD in postweaning pigs when supplemented at 0.5 g/kg in diet. Thus, nucleotide supplementation in combination with reduced dietary protein may have an additive effect in limiting the incidence of PWD in pigs.

The ATTD of DM and GE in the PC and NC groups did not differ; however, it has been previously suggested that reducing dietary CP levels may impair nutrient digestibility in pigs (Xue et al., 2017). This was confirmed by Wang et al. (2024) who reported that 6% reduction in dietary CP (21.5% to 15.5% C.P) reduced ATTD of DM and gross energy in growing pigs. The observed decrease in the ATTD of DM and GE in the 0.1% and 0.3% nucleotides supplemented group aligns with these findings. While the reason for differences in the ATTD of DM and GE between the NC and PC, as well as NC01, NC03, and PC are not entirely clear, our results may indicate that reducing dietary CP may lead to some reduction in digestive capacity, especially as it relates to energy and DM digestibility. Thus, this possibility needs to be taken into consideration when formulating low protein diets for pigs. The overall mechanism of the linear increase in ATTD of N observed in the nucleotide supplemented diets is unclear, but may be related to observed effects of nucleotide in improving gut structure, intestinal barrier function and immune response and increased cell proliferation (Domeneghini et al., 2006; Martinez-Puig et al., 2007; Jang and Kim, 2019; Valini et al., 2021). This result is consistent with Li et al. (2015), who reported that dietary nucleotide supplementation increased the ATTD of N in postweanling pigs. Importantly, it is highly unlikely that these responses are from the extra amino acids in the nucleotide supplement as the quantities of these extra additional amino acids are very minimal in our diets.

Feeding conventional corn-soy diets to pigs provides a sufficient level of CP to fulfill most AA requirements. However, this approach is associated with higher N excretion in feces and urine (Yu et al., 2019). Kohn et al. (2005) established a strong correlation between BUN and urinary N, proposing that BUN is a reliable predictor of N excretion and utilization efficiency in pigs. In this study, concentrations of BUN decreased with a reduction in dietary CP. This finding aligns with a previous study by Yu et al. (2019), who reported that a reduction in dietary CP level resulted in decreased BUN. Furthermore, a study by Yue and Qiao (2008) showed that a linear decrease in dietary CP resulted in a corresponding linear decrease in BUN concentration. Interestingly, nucleotide supplementation further decreased BUN levels, albeit numerically. This additional decrease may indicate improved N utilization efficiency due to nucleotide supplementation. Overall, these findings corroborate the results obtained in the ATTD of N and suggest that pigs fed low CP diets had increased N utilization efficiency, irrespective of nucleotide supplementation. Therefore, in addition to the potential of low protein diets to reduce PWD, these diets may also enhance N utilization, leading to some reduction in nitrogenous waste production and environmental pollution from excessive nitrogen release from pig wastes.

Glutathione (GSH) is crucial for maintaining cellular redox homeostasis, protecting cells from oxidative stress and damage induced by xenobiotic electrophiles (Forman et al., 2009). In the present study, a 6% CP reduction resulted in decreased GSH concentration irrespective of nucleotide supplementation. Hence, diets with reduced CP may decrease antioxidant capacity in postweaning pigs. This result is consistent with a previous study in Huanjiang pigs fed a 14% CP diet that had lower serum GSH levels compared to those fed diets containing 18%-22% CP (Liu et al., 2023). Reduction in serum antioxidant capacity in pigs fed low-protein diets may be consequential during periods of oxidative stress, and this potential negative effect of low-protein diets must be taken into consideration when feeding these diets to pigs.

Serum insulin growth factor-1 (IGF-1) serves as an indicator of metabolism and growth as it plays a pivotal role in promoting normal growth and development in mammals (Jones and Clemmons, 1995). Previous studies reported that a reduction in dietary CP resulted in decreased final body weights and serum IGF-1 concentration in postweaning pigs (Cho et al., 2008; Li et al., 2017). In the present study, serum IGF-1 concentration decreased with reduction in dietary CP, but was partially recovered with nucleotide supplementation at 0.9%, showing a linear increase with higher nucleotide supplementation. This observed trend indicates that an increase in serum IGF-1 concentration by the higher level of nucleotide supplementation may partly explain the observed trend for amelioration of growth performance observed at this level of supplementation. This may represent a novel mechanism of nucleotide effect in regulating pig growth. However, additional studies are required to elucidate the link between nucleotide supplementation and regulation of IGF-1 production.

## CONCLUSION

Results from this study indicate that feeding low-protein diets supplemented with nucleotides after weaning could reduce the incidence of PWD and N excretion, improve protein utilization efficiency, and partially ameliorate the negative effects of low-protein diets on growth performance. However, additional studies are needed to investigate the underlying mechanisms of dietary nucleotide actions.

## Supplementary Material

txae142_suppl_Supplementary_Table_S1

## Abbreviations

ADFI: average daily feed intake
ADG: average daily gain
AOAC: Association of Official Analytical Chemists
ATTD: apparent total tract digestibility
BUN: blood urea nitrogen
BW: body weight
Ca: calcium
CP: crude protein
DM: dry matter
ETEC: enterotoxigenic *Escherichia coli*
FI: feed intake
G:F: gain-to-feed ration
GE: gross energy
GIT: gastrointestinal tract
GLM: general linear model
GLU: glucose
GSH: glutathione
IGF-1: insulin-like growth factor-1
IML: interactive matrix language
INS: insulin
LP: low protein
N: nitrogen
NRC: National Research Council
NUCL: dietary nucleotide
P: phosphorus
PWD: postweaning diarrhea
