# Supplementary material for: Responses in weanling pigs fed low protein diets supplemented with dietary nucleotides
Source: Transl Anim Sci. 2024 Oct 3;8:txae142. doi: 10.1093/tas/txae142 (PMC11497618; doi:10.1093/tas/txae142)
Supplement: txae142_suppl_Supplementary_Table_S1 [file txae142_suppl_supplementary_table_s1.docx]

**Responses in weanling pigs fed low protein diets supplemented with dietary nucleotides**

Abiola S. Lawal, Tobi Z. Ogunribido, Yuechi Fu, Olayiwola Adeola, and Kolapo M. Ajuwon.

**Supplementary materials**

**Supplementary Table S1.** Chemical composition of dietary nucleotides^1^

| Item | Value |
| --- | --- |
| Typical profile |  |
| Nucleotides, % | 6 |
| Crude protein, % | ≥ 50 |
| Moisture, % | ≤ 6 |
| Amino acid profile (g/100 g of product) |  |
| Lysine | 5.5 |
| Methionine | 0.7 |
| Cysteine | 0.4 |
| Threonine | 1.9 |
| Tryptophan | 0.7 |
| Valine | 2.7 |
| Arginine | 2.4 |
| Histidine | 0.9 |
| Isoleucine | 2.1 |
| Leucine | 3.0 |
| Phenylalanine | 6.1 |
| Ileal digestibility (Standardized, in pigs) |  |
| Lysine, % | 95 |
| Methionine, % | 94 |
| Cysteine, % | 65 |
| Threonine, % | 80 |
| Tryptophan, % | 87 |

^1^Nucleosaf^600^ is a premium yeast extract with consistent nucleotides level, obtained by autolysis of proprietary *Saccharomyces cerevisiae* baker’s yeast strains. Purification steps guarantee good product stability while maintaining highly desirable nutrients.
